# A synergistic effect on enriching the Mg–Al–Zn alloy-based hybrid composite properties

**DOI:** 10.1038/s41598-022-24427-8

**Published:** 2022-11-21

**Authors:** Gnanasambandam Anbuchezhiyan, Nabisab Mujawar Mubarak, Rama Rao Karri, Mohammad Khalid

**Affiliations:** 1 Department of Mechanical Engineering, Saveetha School of Engineering, Saveetha Institute of Medical and Technical Sciences, Chennai, 602105 Tamil Nadu India; 2 Petroleum and Chemical Engineering, Faculty of Engineering, Universiti Teknologi Brunei, BE1410, Bandar Seri Begawan, Brunei Darussalam; 3 Graphene & Advanced 2D Materials Research Group (GAMRG), School of Engineering and Technology, Sunway University, No. 5, Jalan University, Bandar Sunway, 47500 Petaling Jaya, Selangor Malaysia

**Keywords:** Energy science and technology, Engineering, Materials science, Nanoscience and technology

## Abstract

Mg–Al–Zn alloys are widely preferred in many applications by considering their excellent properties of high stiffness-to-weight ratio, lightweight, high strength-to-weight ratio, low density, castability, high-temperature mechanical properties, machinability, high corrosion resistance, and great damping. Improving the properties of such alloys is challenging due to their hexagonal crystal structure and other alloying limitations. This study aims to synthesize Mg–Al–Zn alloy by incorporating the alloying elements 8.3 wt% Al, 0.35 wt% Zn on pure magnesium (Control specimen). Then synthesize Mg–Al–Zn/BN/B_4_C hybrid composite by reinforcing B_4_C at three weight proportions (3 wt%, 6 wt%, 9 wt%) along with constant solid lubricant BN (3 wt%) through a stir casting process. The hybrid composite samples were characterized and compared with the performances of the control specimen. The results reveal that 9 wt% B_4_C reinforced samples outperformed through recording the improvement of tensile strength by 28.94%, compressive strength by 37.89%, yield strength by 74.63%, and hardness by 14.91% than the control specimen. Apart from this, it has reduced the corrosion area (37.81%) and noticed negligible changes in density (increased by 0.03%) and porosity (decreased by 0.01%) than the control specimen. The samples were characterized using SEM, XRD, and EDAX apparatus.

## Introduction

Low-density materials are becoming more prevalent in automotive, aerospace, and marine applications due to their less dense and higher energy efficiency. Compared with other metals and alloys, magnesium and its alloys have gained interest due to their less dense performance and high compressive strength. Further, magnesium is recyclable with a reduction in the emission of CO_2_ is another important reason for fulfilling functional applications^1^. Despite their excellent physical properties, these materials have limited applications due to low strength, modulus, and wear resistance, are highly reactive, and have poor creep resistance at high temperatures^2^. These disadvantages can be removed by adhering to desired processing methods and adding alloying elements or reinforcement^3^. Based on the presented results, ceramics such as Silicon carbide, Aluminium oxide, Boron carbide, Silicon nitride, Titanium dioxide, Aluminium nitride, Titanium nitride, Yettrium oxide, and Titanium carbide have been used to strengthen particulates that are composed of magnesium composites^4^.Ceramic reinforcements can be encapsulated with a matrix material, leading to limitations. Increasing the weight fraction of unique ceramic reinforcing particles in the matrix material increased hardness, density, toughness, and brittleness, but decreased ductility and elongation percentage was observed^5^. This is due to the matrix alloy’s homogenous distribution of strengthening particulates, while agglomeration results in inferior properties^6^. The literature studies determined that the inclusion of the secondary ceramic particulate in the parent material strengthens the material through a reduction in grain size, determines the mechanical properties of composites, and is affirmed as a hybrid composite. Numerous studies have been conducted on synthesizing magnesium hybrid composites using different processing methods and reinforcements^7^. The powder metallurgy approach was used to characterize the worn performance of magnesium composites. The inclusion of Graphite extended the wear resistance of hybrid mixtures and reduced the microhardness properties^8^. The semisolid stirring method was employed to develop the dynamic tensile behaviour of magnesium hybrid nanocomposites. It was observed that the strain rate hardening was distinct at different temperatures when Nanoscale SiC and MWCNT were used for reinforcement^9^. The inclusion of SiC particles effectively improved the wear rate of synthesized composites due to the reinforcement of short fibres and cast composites using the squeeze casting method^10^. Liquid metallurgy has been used to develop boron carbide’s mechanical properties and graphite’s toughened magnesium intermix complexes. Incorporating graphite into matrix alloy results in a decrease in wear characteristics^11^. Microstructure and physical properties of aluminium hybridized composites were examined using titanium diboride and boron nitride as strengthening parts, with the inclusion of BN having the primary purpose of enhancing wettability while enhancing wear resistance^12^. Based on the literature, it has been concluded that the density has significantly influenced the choice of reinforcement for synthesizing magnesium alloy hybrid composites. If otherwise, the density of such combinations would increase and be mismatched with the weight reduction properties for functional applications^13^. As part of the proposed innovative study, ceramic reinforcements with low-density materials have been chosen for the continued development of magnesium alloy hybrid composites. A literature study was performed for the explicative research gap, and some implications for this study were summarized. Mg–Al–Zn alloys have a hexagonal crystal structure, which affects fundamental properties such as toughness, flexibility and other properties. Aside from that, the surface energy of this material is high compared to other lightweight materials such as aluminium or zinc. Still, it has less corrosion and wear resistance than aluminium. It was also observed that in magnesium alloys, 10 wt% Al improves tensile strength, hardness, and castability by increasing solid-solution strengthening, and 0.35% Zn forms MgZn_2_ phases along grain boundaries, resulting in excellent age hardening and found to provide improved characteristics. However, adding alloying elements is restricted to the base magnesium alloy, as it is integral to the material's environmental compatibility^14^. It was nailed that only the addition of ceramic reinforcing particles such as borides, carbides and nitrides improve the properties of Mg–Al–Zn alloys. Further investigation into the wettability of boron carbide and boron nitride with Mg–Al–Zn alloys as particulate-strengthened particles for consolidating hybridized composites was found to be lacking. Since magnesium exhibits highly reactive and forms magnesium oxide when exposed to the atmosphere, it is a major drawback of such alloys. It was forecasted that by adding low density of reinforcement to a combination of different base materials, the density of composites was restored, and its mechanical properties were notably improved^15^. A literal analysis found that the inclusion of B_4_C reinforcement of different particle sizes results in a higher mechanical strength of the material. Still, BN reinforcement is limited despite having a lower density than B_4_C^16^^.^ It also inferred that boron carbide and boron nitride had not been extensively investigated as particulate-strengthened particles for consolidating Mg–Al–Zn alloy-based hybrid composite. The particulates recommended for reinforcement have a lower density of 2.5 g/cm^3^ and 2.1 g/cm^3^ for boron carbide and boron nitride than other ceramic reinforcements. By adding this combination of reinforcement in different base materials, the density of the final material (composite) has been restored, and its mechanical properties further improved. The effects strengthen the properties of Mg–Al–Zn alloy (91.35 wt% pure magnesium, 8.3 wt% Aluminium, 0.35 wt% Zinc) by reinforcing B_4_C at three levels (3 wt%, 6 wt%, 9 wt%) with constant solid lubricant BN of 3wt%. was not yet reported so far. Since BN is lamellar in a structure like molybdenum disulfide and graphite, and comparison to these, it is a better solid lubricant. Due to this factor, BN is preferred as secondary reinforcement, and its percentage weight is kept constant in the present investigation. Hence this piece of research addresses synthesizing, characterizing and testing of Mg–Al–Zn/BN/B_4_C hybrid composites samples and comparing their performance with synthesized as cast Mg–Al–Zn alloy (control specimens).

The novelty of the present study is synthesizing Mg–Al–Zn alloys by incorporating the alloying elements 8.3 wt% Aluminium, 0.35 wt% Zinc into pure magnesium. Then, this casted alloy was strengthened with hard ceramic strengthening particles of B_4_C (3 wt%, 6 wt%, 9 wt%) with solid lubricant BN (3wt%) using the bottom pouring stir casting process and compared the three Mg–Al–Zn/B4C/BN hybrid composites performances with Mg–Al–Zn alloy in terms of density, porosity, hardness, tensile strength, yield strength, percentage of elongation, compressive strength and corrosion rate, including microscopic investigations like SEM, XRD etc., for ensuring the quality of new material for marine applications such as engine casings, hulls and fins.

## Materials and methods

### Matrix and reinforcement

This work uses commercially available alloys of Mg–Al–Zn–Si–Ni to develop magnesium alloy, and the chemical constituents of these materials are outlined in Table 1. Including aluminium and zinc in magnesium alloys increases their hardness and strength at room temperature. It is believed that Mg–Al–Zn alloys are most commonly used for weight deduction at room temperature. Further, it has a high strength-to-weight ratio, good flexibility, better damping properties, and excellent castability. It is ideal as a matrix material for fabricating magnesium hybrid composites^17^. The reinforcements B_4_C of (~ 1 µm) and BN (< 10 µm) of particle size from Sigma Aldrich are also used to incorporate such composites.

**Table 1:** Empirical constituents of Mg–Al–Zn contamination^18^.

Elements	Al	Mn	Zn	Si	Cu	Ni	Mg
% composition	8.3	0.13	0.35	0.5	0.5	0.03	Remaining

### Stir casting method

**Figure 1:**
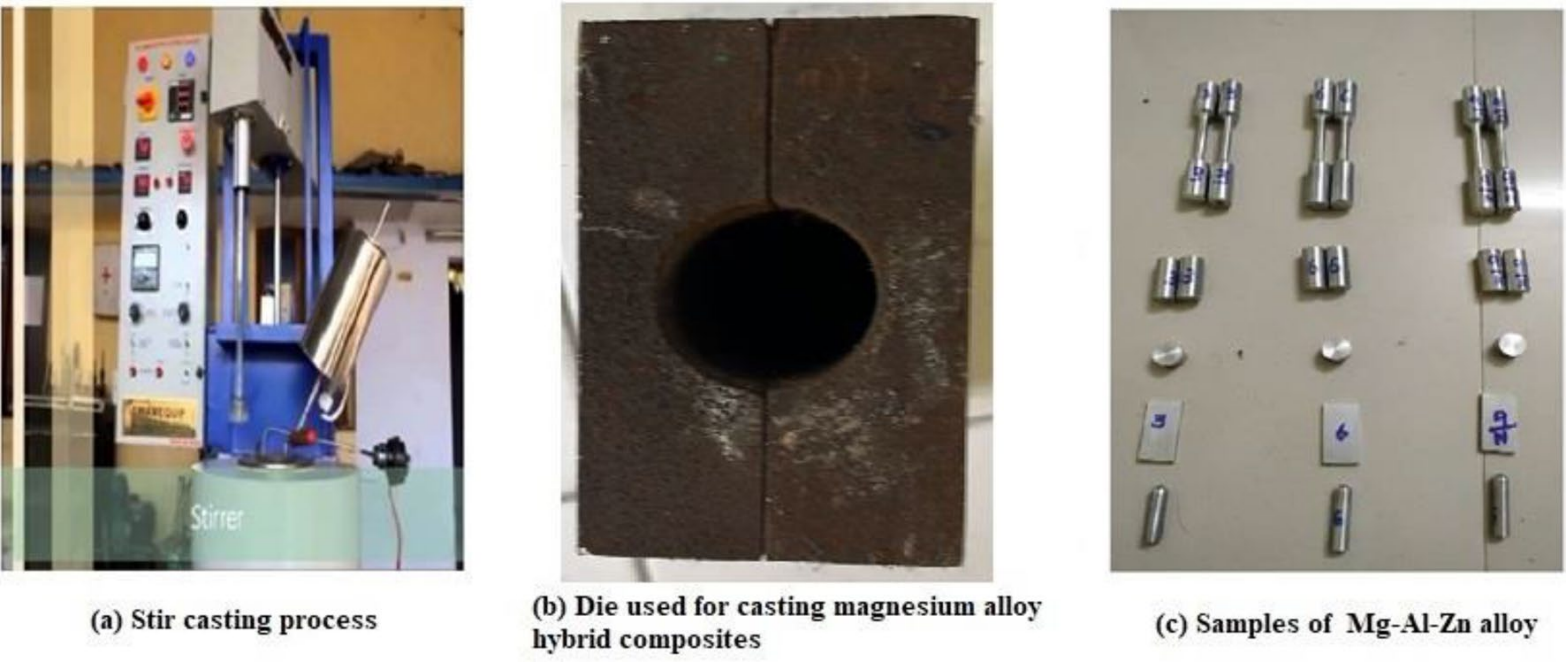
(**a**) Stir casting apparatus (**b**) Die used for synthesizing magnesium alloy hybrid composites (**c**) Samples of Mg–Al-Zn alloy.

The Stir casting apparatus of the bottom pouring type is used to produce magnesium alloy hybrid composite materials with an inert gas environment, as shown in Fig. 1a. The die used for casting magnesium alloy hybrid composites is shown in Fig. 1b, and the fabricated samples of Mg–Al–Zn alloy composites are presented in Fig. 1c. To make magnesium melt, the resistance heating furnace is initially preheated to 250 °C, followed by placing the required amount of casted magnesium alloy in the furnace and melting it for 45 min before the temperature is raised to 750 °C. Through external spruce, the reinforcement of B_4_C particles of varying their weight percentage (3wt%, 6wt%, and 9wt %) and keeping constant of BN (3wt %) is added into the melt. To prevent oxidation and burning, 3.5 l/min of a CO_2_ and SF_6_ mixture was allowed to discharge into the furnace, and the melting temperature was raised to 750 °C. It was also noted that density is important in enhancing the even dispersion of ceramic particles within the base material^9^. The reason for that is that if the density of reinforcement is minimal, it will remain alone at the top of the molten slurry, whereas if the density is maximal, it will settle down to the bottom. By using an effective process parameter, this problem can be resolved.

For effective stirring, the stirrer diameter must also be considered. The solid particles remain suspended at the outside edge of the vessel when the stirrer diameter is too small and can remain in the vessel’s centre if it is too large. As a result, the stirrer diameter has been set to 0.4D, per the literature citation^19^. A parameter associated with the stir casting method must be considered to achieve homogenous particle distribution in the matrix alloy. Due to this, the stirring speed and stirring time were maintained at 600 rpm and 15 min, as cited in the literature^20^. It was found that pouring temperature significantly affects particle distribution. Higher melt temperatures tend to float the ceramic particles to the surface of the melt, while lower melt temperatures reduce melt viscosity and make casting more difficult^21^. Owing to these facts, the optimal temperature for casting magnesium alloy hybrid composites was maintained at around 700 °C for pouring the molten slurry into the die cavity. An EN24 die with a diameter of 0.022 m and a length of 0.2 m is placed in the bottom of the apparatus and coated with sulfur powder to prevent oxidation and burning of the molten slurry. Then, the molten slurry is poured into the die and left to cool at atmospheric pressure in the die alone. The same procedure has been repeated using different reinforcement weight percentages and synthesizing magnesium alloy hybrid composites.

The increase of low-density ceramic particulates (B_4_C) up to a maximum of 12 wt% in molten magnesium alloy cause agglomeration even when stirring is applied to obtain a homogeneous distribution of such particulates in the matrix. This cannot be achieved because a few unwetted ceramic particles that float on the melt surface will stick together and accumulate in one region, giving it poor mechanical properties. Utilizing a higher percentage of ceramic particulates in the cast magnesium composite increases its porosity, forming a SiO_2_ layer on top of the ceramic particulates as they are filled with molten slurry. It was noted that when the number of reinforcing particles increased to the maximum, the percentage of clusters increased along several regions of the base material leading to pores and a gas layer surrounding the ceramic particles causing clusters to float. As a result, the weight percentage has been limited to 9wt%, and the results of the synthesized hybrid mixtures were discussed in Xiang et al.^22^.

### Performance measures

Performance measures for magnesium alloy hybrid composites were prepared using ASTM standards. A De-Winter trinocular inverted metallurgical microscope is used to characterize the microstructure of synthesized composites. The density of synthetic composites has been calculated using Archimedes principles with ethanol as suspending medium. Using Wilson Wolpert Germany, a micro Vickers hardness test is used to determine the microhardness of materials under a 1 kg load. Using a universal testing machine, the ASTM E8 standard is used to study the tensile behaviour of hybridized mixtures. The maximum load range is 10 tons, and the shear rate is 0.5 m/min. For evaluating the compressive strength of an intermixture at room temperature, the ASTM E9 standard is used. The corrosion resistance of hybridized composite composites has been analyzed using the B117 salt spray test^23^.

## Results and discussion

### Microstructure of Mg–Al–Zn alloy hybrid composites

**Figure 2:**
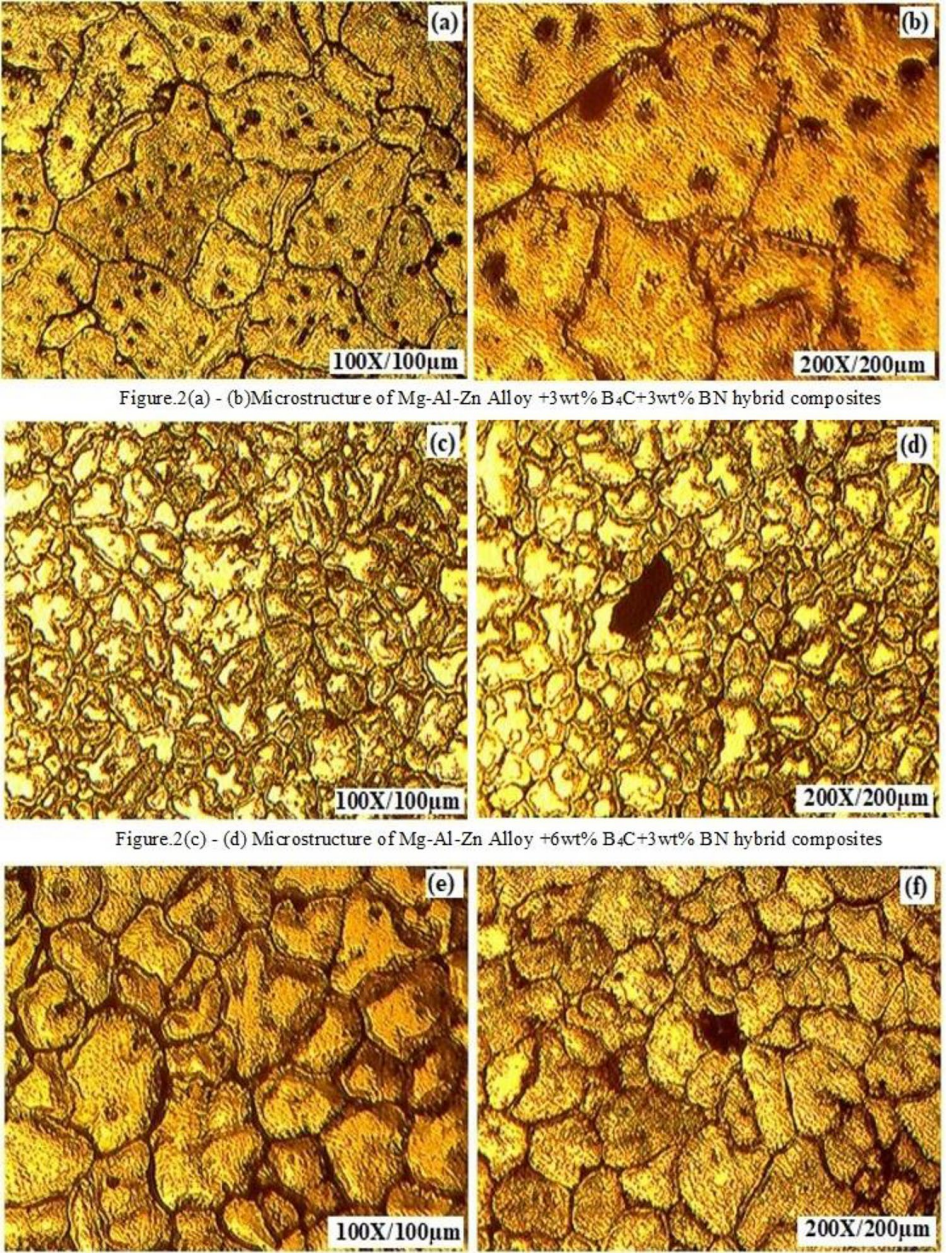
(**a–f**). Microstructure of Mg–Al-Zn Alloy composite strengthened with boron carbide of varying weight proportions and boron nitride constant.

An optical microscope is used to characterize the finely structured magnesium alloy hybrid composites. Figure 2a to Fig. 2f examine the as-cast and etched hybridized composites with differing percentages of boron carbide and boron nitride by weight. Picral is used as the etching agent^24^^.^ The microstructure of both B_4_C and BN reveals the homogenous distribution of strengthening particles without any evidence of a cluster. Moreover, the higher inclusions of B_4_C particulates in the parent material display the primary magnesium grains and appear finer. Due to impurities, grain boundaries of newly synthesized hybrid composites show microparticles of eutectic precipitates. Boron carbide has a larger granularity than boron nitride, so the distribution of BN is leaned and appears as dull shiny white particles due to the inferior inclusion of BN in the matrix alloy. It was presumed that the microstructures of the synthesized magnesium alloy hybrid composites possessed B_4_C, Mg, MgO and MgB_2_ interphases. This is because increasing the proportion of boron carbide increases the formation of the MgO and MgB_2_ phases due to the heating process and reactions between the immixtures^24^. The microstructural studies found that good interfacial integrity between the Mg matrix and the hybrid ceramic reinforcement was esteemed regarding the nonappearance of voids and debonding at the particle–matrix interface. This stimulates the enhancement of the mechanical properties of synthesized magnesium alloy hybrid composites, as inferred in similar findings^25^^.^

**Figure 3:**
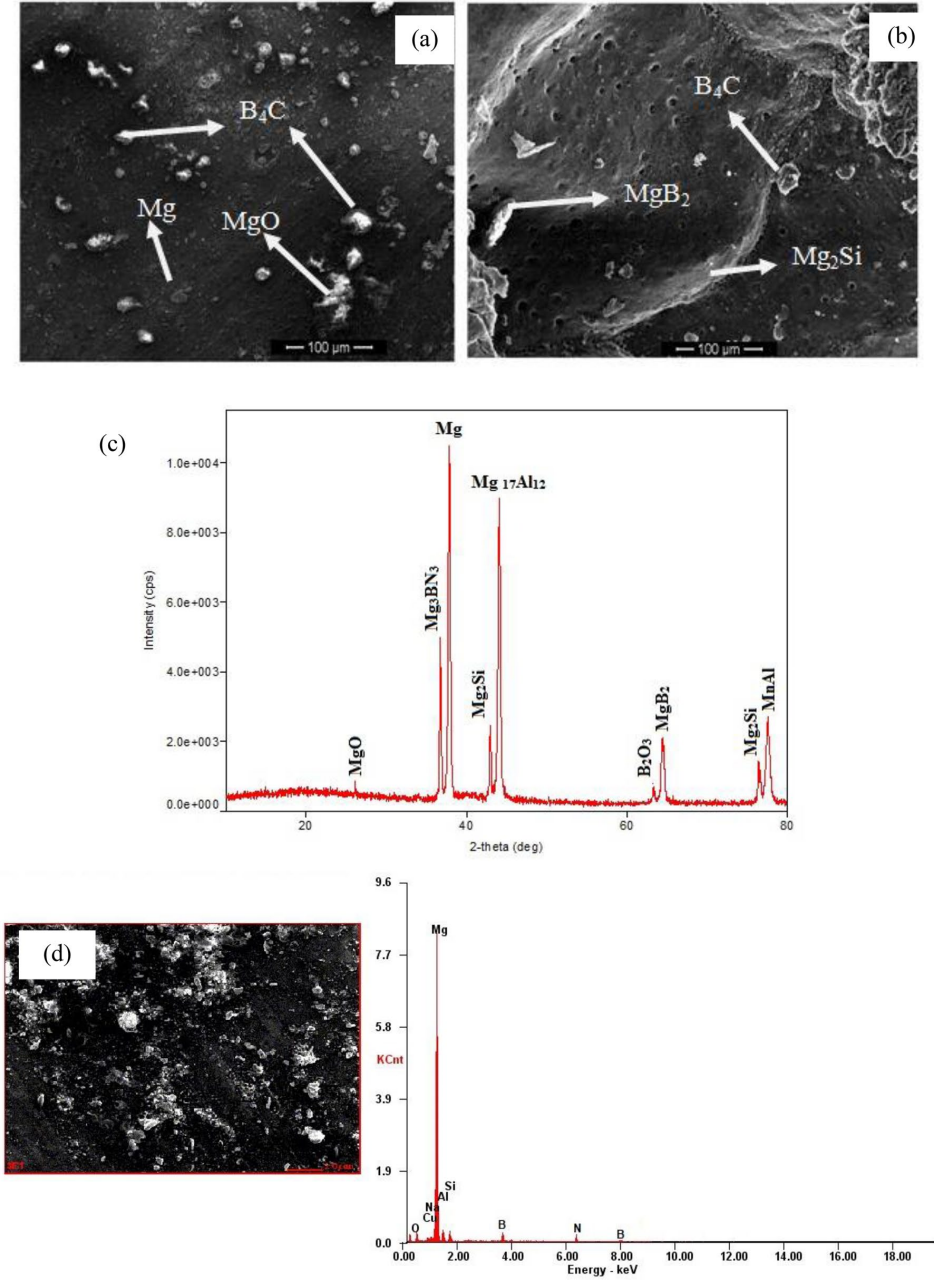
SEM image of Mg–Al–Zn Alloy composite strengthened with (**a**) 6wt% boron carbide and 3% boron nitrate, (**b**) 9wt% boron carbide and 3% boron nitrate, (**c**) XRD image of Mg–Al-Zn Alloy composite strengthened with 9% boron carbide and 3% boron nitrate, (**d**) EDAX analysis of the image of Mg–Al-Zn Alloy composite strengthened with 9% boron carbide and 3% boron nitrate.

The morphology characteristics of magnesium alloy hybrid composites and the distribution of ceramic reinforcement particles are analyzed using SEM, as shown in Fig. 3a,b. It was determined that B_4_C appears as a needle-like structure within the magnesium alloy matrix which is constantly dissipating through regions of grain boundaries and internal grain boundaries. Because of the pinning effect, BN acts as nucleation sites and reduces grain size while causing a reduction in grain growth due to the higher proportion of grain boundary particles in the matrix alloy^26^.

The interfacial reaction plays a vital role in increasing the mechanical properties of magnesium hybrid composites. The presence of boron carbide interacts with Mg–Al–Zn magnesium alloy and forms the intermetallic components of Mg_17_Al_12**,**_ MgB_2_, Mg_2_Si, Mg_3_BN_3_, MgO, B_2_O_3,_ MgC_2_ as shown in Fig. 3c as inferred in the literature^27^. In the XRDA, two major principal peaks are observed in the intermixture, such as Mg and B4C, which resulted in small molecules of MgO being formed by the partial reaction of Mg with oxygen. In contrast, MgC_2_ is formed by reaction with free carbon in the system, but this phase is unstable and rapidly deteriorates^28^. Also, it was observed that molten magnesium reacts with B_4_C to form magnesium diboride, which liberates elemental carbon. The presence of MgB_4_ peaks in the XRD pattern proved that MgB_2_ partially decomposes to form MgB_4_, which agrees with similar findings^29^. It was also found that it accommodates heterogeneous nucleation sites during solidification, hence reducing grain size in the matrix alloy. This acts as a strengthening mechanism of fabricated magnesium alloy intermixture^30^.

The EDAX analysis of the Mg–Al–Zn alloy hybrid intermixtures shown in Fig. 3d indicates that the major elements are Mg, Al, B, Si, N, and some oxides. Observations have shown that particle Si in a matrix continues to react with Mg to form Mg_2_Si^31^. As a result of the presence of Mg_2_Si, the mechanical properties of the developed Mg–Al–Zn alloy immixture are enhanced, and this compound contains a mixture of Mg–Al–Si–Mn–B–N compositions.

### Assessment of density and porosity of magnesium alloy intermixture

It seems important to measure the density of synthesized magnesium alloy hybrid composites because they contain ceramic strengthening particles with a density considerably higher than the base material. A higher density of magnesium alloy reinforcement will result in a higher density of synthesized composites. This has detrimental effects on the perception of weight reduction properties. Considering those factors, the density and porosity of high-density magnesium alloy hybrid composites have been measured using the rule of mixture equation. In contrast, their experimental density has been measured using Archimedes' principle with ethanol as a suspending medium. Eqs. (1) and (2) give a formula for calculating magnesium alloy hybrid composites' theoretical density and porosity^32^^.^

1 $$Theoretical\, density=(\rho \,of \,AZ91\times wt\mathrm{\% }\,of\,AZ91)+ (\rho\, of \,reinforcements\times wt\mathrm{\% }\,of \,reinforcements)$$

2 $$\% \,Porosity=\frac{Thoretical\, density-Experimetal \,density}{Thoretical \,density}\times 100$$

**Figure 4:**
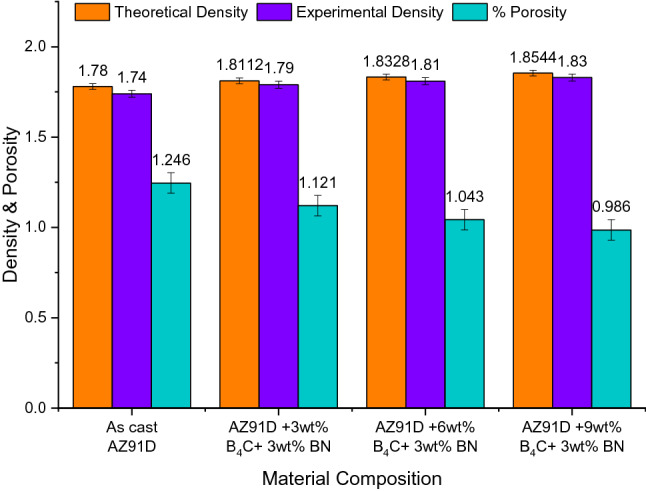
Density and porosity of Mg–Al–Zn alloy hybrid composites.

Owing to the accession of superior density particulates in the heterogeneous mixture, strengthening in the proportion of reinforcement, and the existence of interfacial reaction between the matrix and reinforcement, the density of the Mg–Al–Zn alloy hybrid is increased substantially to a minimum of 0.038% compared with monolithic magnesium alloy as inferred in Fig. 4. Increasing the percentage of binary ceramic particulates significantly reduces the porosity of actualized composites^33^. Moreover, ceramic strengthening particles added to the molten alloy caused the particles to accumulate at the melt surface, even after mechanical stirring mixed the reinforcement particles^29^. The majority of these particles tried to stick together once the stirring stopped. However, the reduced viscosity of these particles caused them to remain in the matrix area, which resulted in a uniform dispersion throughout the matrix. This is due to a reduction in the entrapment of gas particles while adding the hybrid reinforcement particles in the molten melt and minimization of the shrinkage effect at the time of solidification. This contributes to the increased compressive properties of developed magnesium alloy hybrid composites.

### Hardness

**Figure 5:**
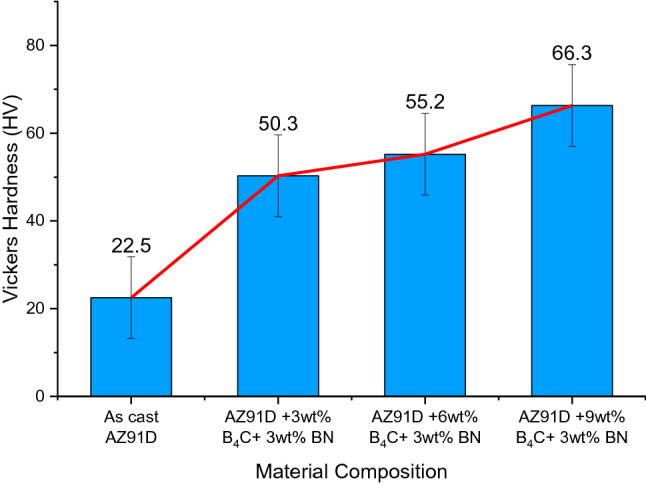
Hardness of magnesium alloy hybridized composites.

The hardness of hybrid composites developed by varying the proportion of B_4_C and keeping the BN reinforcement is shown in Fig. 5. Compared with monolithic magnesium alloys, it is shown that the addition of ceramic reinforcement increases the hardness of hybrid composites to an optimum of 14.91%. This is due to factors such as the uniform distribution of ceramic particulates inside the parent material, the presence of hard interface particles, and the large difference in thermal expansion coefficient between the matrix and reinforcement that significantly increased the dislocation density in the microstructures of synthesized composites as reported in the literature^34^. Furthermore, the combination of ceramic strengthening particulates in the magnesium alloy matrix increases boron carbide throughout the regions and reduces the matrix grains. Consequently, a large increase in grain boundaries, which act as obstacles to dislocations, causes an increase in the microhardness of synthesized hybrid composites, as inferred in the literature^35^. In addition, the inclusion of secondary reinforcement solid lubricant (BN) assists in increasing the hardness of the synthesized composites and acts as a barrier, along with the B_4_C, in preventing the passage of dislocation during indentation.

### Tensile and compressive strength

**Figure 6:**
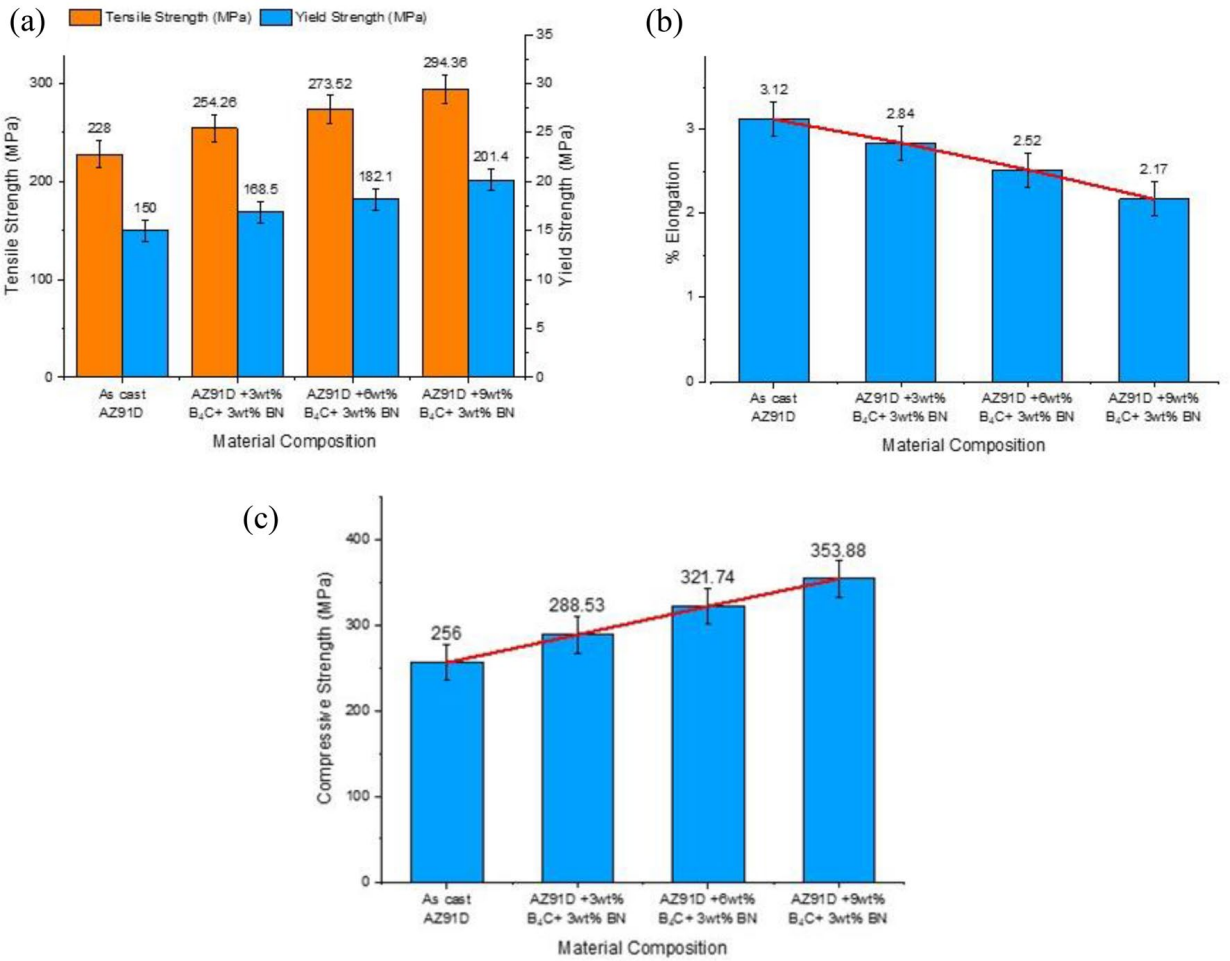
(**a**) Tensile and yield strength of Mg–Al–Zn alloy hybrid composites (**b**) Percentage elongation of Mg–Al–Zn alloy hybrid composites (**c**) Compressive strength of Mg–Al–Zn alloy hybrid composites.

In this study, it has been observed from Fig. 6a that by enhancing the proportionality of hard ceramic particulates, the ultimate tensile and yield strengths of the magnesium alloy heterogeneous mixture increased considerably. Still, the elongation percentage decreased, as Fig. 6b inferred.

This occurred due to an excellent load transfer from matrix to ceramic phase, Orowan strengthening mechanism, grain refinement, and differences between the thermal and elastoplastic performance of the synthesized hybrid composites^36^. When the reinforcement is uniformly distributed in the magnesium matrix, it exhibits high-density dislocations. It, therefore, causes a discrepancy between the parent material and reinforcement particles, which acts as a strengthening effect when hybrid composites are strained^37^.

A compressive strength test was conducted as per ASTM E9 for magnesium alloy hybrid composites^38^. As discussed in the literature, under loading conditions, magnesium matrix materials' deformation varies for tensile properties (slip) and compressive properties (twinning)^39^. In contrast with Mg–Al–Zn base alloys, the compressive strength of fabricated magnesium alloy mixtures is significantly enhanced, as shown in Fig. 6c. This leads to an interfacial strength distribution throughout the magnesium matrix, a reduced twinning movement, and a refined grain structure. Observations have shown that matrix grain refinement in the Mg matrix is affected by the reinforcement and particle distribution, indicating that the uniformity of particle distribution is sufficient for grain size reduction in a hybridized magnesium alloy mixture. It is also inferred that during straining, the inclusion of secondary reinforcement would alter the strain rates of magnesium alloy hybrid composites; therefore, the fracture surface appears smooth. A heterogeneous deformation on the magnesium alloy matrix occurs at the grain boundary and, as such, inhibits plastic deformation but leaves the crystalline core of the grain when compressive loading occurs; hence the compressive properties of synthesized magnesium alloy hybridized composites significantly increase, as cited in the literature.

**Figure 7:**
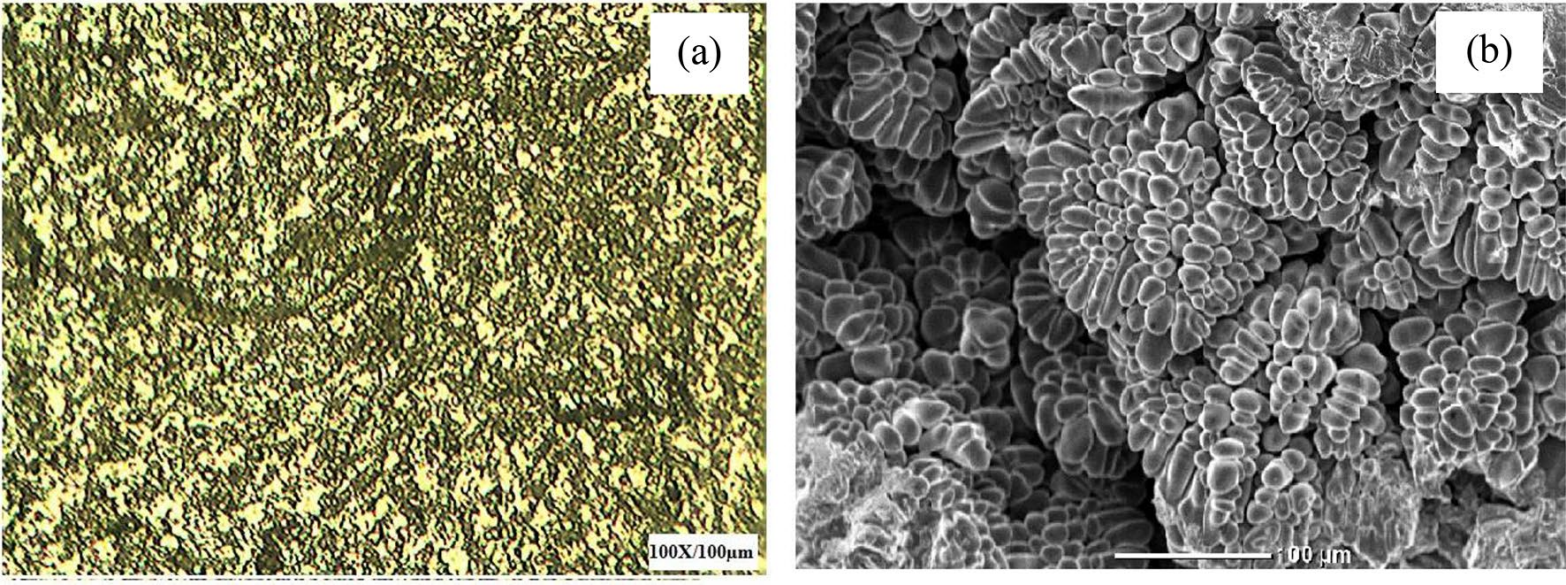
(**a,b**) Grain refinement of AZ91 with 9wt% boron carbide and 3wt% boron nitrate.

It has also been noted that the increased strength at room temperature is due to enhancing grain refinement, as shown in Fig. 7a,b. By increasing the hardening of matrix alloy reinforcement, crack propagation has been minimalized to its maximum when applied loads to composites. Additionally, the deformation mechanism contributes significantly to enhancing the tensile properties of magnesium alloy hybrid composites because adding aluminium and zinc elements results in less stacking fault energy reduction than among the other elements^34–36^^.^ Furthermore, incorporating secondary reinforcement (BN) can lead to the generation of higher heterogeneous nucleation sites (Mg_17_Al_12_) and consequently lead to the greater fracture toughness of the magnesium hybrid composites. As a result, premature failure occurs if the bonding between the matrix and reinforcement is not substantial. This cannot be observed in synthesized composites.

Furthermore, it is found that when the percentage of reinforcement is increased, the percentage of elongation decreases significantly. Consequently, the composite may be brittle because of the lack of superplasticity produced by including hard ceramic reinforcement. It is also noted that particles forced against each other during solidification restricted the growth of the primary phase and nuclei of the AZ91D alloy.

### Corrosion resistance of magnesium alloy hybrid composites

Magnesium alloys are highly reactive in humid or wet environments due to their loosened oxide layer, which causes them to corrode much more easily in marine environments with high aqueous concentrations^40^^.^ The occurrence of galvanic corrosion in magnesium alloys can also pose a problem since magnesium has the lowest electrode potential and functions as an anode. Even though alloying Al and Zn with Mg increase their strength, they are more prone to galvanic corrosion. In corrosion tests performed on Mg and AZ91 alloys using 0.1 M NaCl solutions, the AZ91 alloy displayed higher corrosion than Mg metal. The influence of alloying elements like Zr and Al on the corrosion resistance of Mg was tested electrochemically. They found that AZ91 alloy shows the worst corrosion resistance (9E^−4^ mm/year) among AZ31, AZ91, AM60 and ZK60 alloys^40^. During immersion of magnesium alloys in NaCl solution, the following reactions occur on the surface as illustrated in Eqs. (3, 4 and 5), and a similar finding was inferred in the literature^41^.

3 $${\text{Mg}} \to {\text{Mg}}^{{{2} + }} + { 2}^{{{\text{e}} - }}$$

4 $${\text{2H}}_{{2}} {\text{O }} + {\text{2e}}^{ - } \to {\text{H}}_{{2}} + {\text{ 2OH}}^{ - }$$

5 $${\text{Mg}}^{{{2} + }} + {\text{ 2OH}}^{ - } \to {\text{Mg}}\left( {{\text{OH}}} \right)_{{2}}$$

When magnesium comes in contact with an aqueous solution, magnesium hydroxide is formed on the magnesium surface, serving as a protective layer. However, since it is porous, this layer will not protect the alloy from corrosive mediums containing Cl. Suitable ceramic strengthening particles can be added to magnesium alloy to overcome this problem.

**Figure 8:**
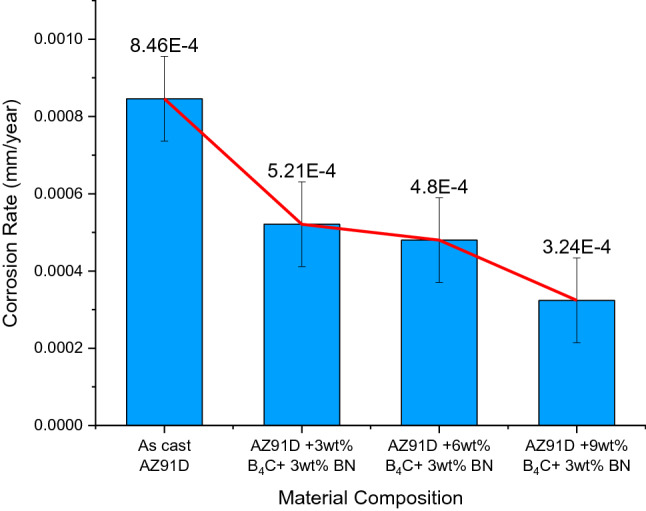
The corrosion resistance of magnesium composites.

The developed hybrid composites’ corrosion resistance was evaluated according to the ASTM-B117 salt spray test, and the results are shown in Fig. 8. Adding hybrid reinforcement (B_4_C and BN) to magnesium alloys increases corrosion resistance due to galvanic coupling, interfacial phase formation, and microstructural changes between the reinforcements and matrix. Further, the corrosion rate of synthesized composites displays a two-phase microstructure consisting of a crystalline magnesium matrix with an aluminium phase (Mg_17_Al_12_) over the grain boundaries^42^. Hybrid composites containing eutectic phases such as Mg, Zn, and Cu show enhanced corrosion behaviour in a corrosive solution of 3.5% NaCl, which affects the volume, composition, and distribution of other eutectic phases.

**Figure 9:**
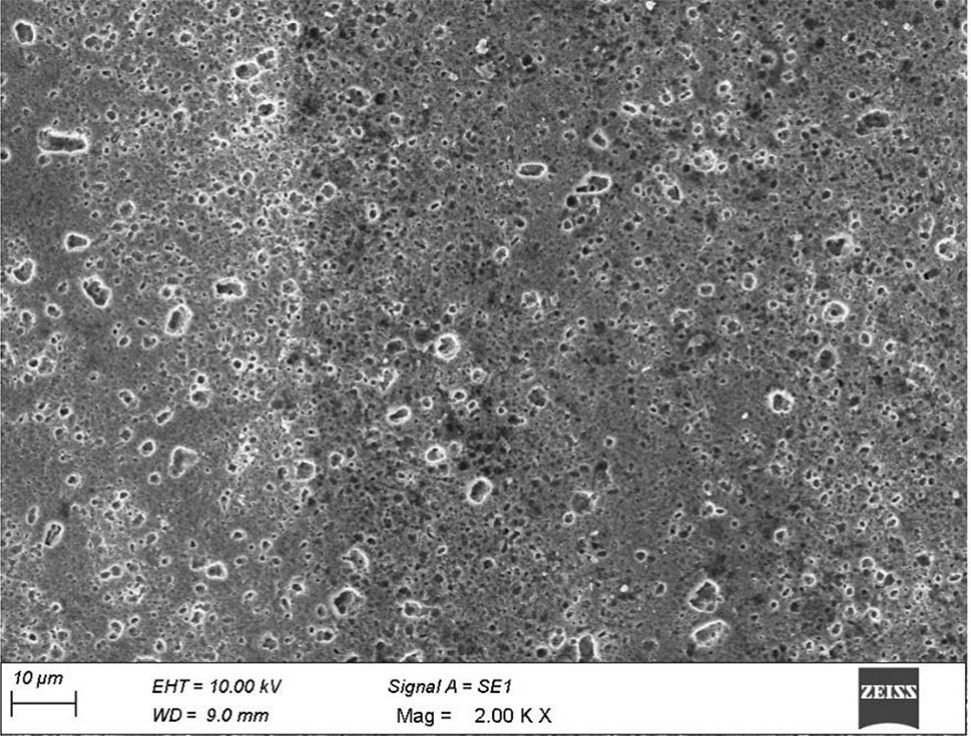
Morphology of various phases of AZ91D + 9wt% B_4_C + 3wt% BN.

 In an investigation of a hybrid composite, it has been determined that the alpha-Mg matrix, which acts as a micro cathode, stimulates corrosion at micro-scales, as found in an alpha-Mg matrix as cited in the literature^43^. The presence of the β phase reduces the reactive surface of hybrid composites, and, as a consequence, the amount of corrosion observed on their surfaces has been reduced. Furthermore, for each alpha grain boundary, a continuous beta phase hinders the corrosion from dispersing from one alpha grain to another, preventing corrosion from forming on the surface of α grains. Thus, the magnesium alloy hybrid composites get dissolved, and the full beta film is exposed to the solution, significantly increasing corrosion rates. From the SEM image, it is evident that the β phases appear intense. The α phase materializes finer due to its occurrence of solidification immediately at room temperature, as shown in Fig. 9. In addition, the appearance of the β phase appears narrow and continuous, thereby inhibiting the corrosion rate of the α phase uniformly through the corrosion that forms on the composites externally. It has been observed that, upon rapid cooling of these composites, they form primary grains with an irregular eutectic of α and β phase; thus, they exhibit enriched forms of eutectic of α, which limit the probability of the occurrence of essential α grains. A grain size reduction has been observed due to increasing solution concentration relative to initially formed internal α grains. The presence of fine grains is confined to being stable β phases. Fine grains are confined to being stable β phases over α grain boundaries and react as an obstacle to giving the corrosion moment an increased value, consequently decelerating.

Based on the experimental studies, B_4_C/BN reinforced Mg–Al–Zn alloy synthesized using the stir casting method significantly improved due to enhanced wettability between the immixtures. Similar findings using those as reinforcement are discussed and compared in Table 2.

**Table 2:** List of Findings similar reinforcement in literature.

Ref no.	Description	Findings
^44^	Al7075 reinforced with (3%, 6%, and 9%) of B4C while maintaining a constant (3%) of BN	Using boron carbide at 6% and 9% reduced corrosion by 22.4%. The corrosion rate was reduced by 18.5% with the addition of 3% and 6% boron carbide
^45^	Al6061/B4C/BN (2, 4, 6, and 8 wt%) B4C as well as 2% BN	8 wt% B4C reinforcement, an erodent discharge rate of 8 gmin^-1^, an erodent velocity of 80 ms^-1^, and an impingement angle of 45°
^46^	Al7075/B4C/BN) (3%, 6%, and 9% of B4C while maintaining a constant BN of 3%	The ultimate tensile strength, yield strength, and hardness are increased by adding B4C (9%) and BN (3%) and are 57, 44, and 72% greater than those of the unreinforced aluminium matrix. The density of the final composite rose significantly than the matrix material
^47^	Equal amounts of B4C and BN nanoparticles in an Al 7010 matrix at weight percentages of 0, 0.5, 1, 1.5, 2, and 2.5	Up to a concentration of 2 wt% of nanoparticles, microhardness increases; after that, it decreases. Improved friction coefficient by 2.5wt%; reduced wear rate
^48^	AA6061 alloy reinforced hybrid composites with 5–15 weight percent h-BN and 5–15 weight percent boron carbide	The 15% B_4_C and 5% h-BN hybrid composite achieved a considerable increase in tensile strength up to 82% compared to the AA6061 matrix alloy Due to the comprehensive lubricating action on the entire worn surface, B_4_C 10wt% and 10 wt% h-BN demonstrated improved wear resistance
^49^	Aluminium 7010 reinforced with 0 to 2.5 percent of boron carbide (B_4_C) and boron nitride (BN) nanoparticles mixed in equal parts as reinforcing materials	With an increase in particle reinforcement up to 2 Wt% and then increased, the composite’s percentage of elongation and impact strength is decreased. As the weight percentage of B_4_C and BN particles rose to 2, the tensile strength and microhardness dropped
^50^	Al6061/B_4_C/BN (2, 4, 6 and 8 wt%) B_4_C as well as 2% BN	B_4_C 8% reinforcement showed very little wear

The results of relevant and recent investigations were reviewed and presented in Table 2. It is found that the B_4_C and BN reinforcements significantly modify the properties of the materials. The present investigation focuses on improving the properties of Mg–Al–Zn alloy with B_4_C and BN reinforcements. From the experimental results, it is observed that Mg–Al–Zn alloy reinforced with 9 wt% of B_4_C and 3 wt% of BN improved hardness by 14.91%, compressive strength by 37.89%, yield strength by 74.63%, tensile strength by 28.94%, and corrosion resistance by 37.81%. It is observed that there are negligible changes in the density and porosity, which are increased by 0.03% and decreased by 0.01%, respectively.

## Conclusions

This study aims to strengthen Mg–Al–Zn alloys using B_4_C and BN as ceramic-strengthening particulates. The Mg–Al–Zn alloy (91.35 wt% pure magnesium, 8.3 wt% Aluminium, 0.35 wt% Zinc) was cast and further strengthened; a stir casting method was used utilized to synthesize three kinds of Mg–Al–Zn/B_4_C/BN hybrid composites. Among the developed hybrid mixture, 9 wt% B_4_C/BN reinforced Mg–Al–Zn alloys hybrid composite exhibited the optimum results. Morphological studies ensured that there was good interfacial integrity between the Mg matrix and the hybrid ceramic reinforcement, as no voids or deboning occurred at the particle–matrix interface. It was also affirmed that the reduction in grain size in the matrix alloy was identified and ensured that they act as a strengthening mechanism of fabricated magnesium alloy intermixture. From the XRDA analysis that the presence of intermetallic components of Mg_17_Al_12_, MgB_2_, Mg_2_Si, Mg_3_BN_3_, MgO, B_2_O_3_, MgC_2_. EDAX analysis indicates that this hybrid intermixture blends Mg–Al–Si–Mn–B–N compositions, indicating improved mechanical properties. The significant outcomes of mechanical, corrosion, and hardness properties are furnished below.

- Due to the presence of the Mg_2_Si interface and an increment in the proportional of strengthening particulates, the denseness of synthetic immixture substantially amplified to a minimum of 0.03%, and a reduction in porosity has been observed as 0.01% compared with monolithic magnesium alloy.
- The mechanical properties such as hardness (14.91%), tensile strength (28.94%), and yield point (34%) of synthesized composites are significantly increased. On the other hand, the percentage elongation has decreased compared to unreinforced alloy.
- The compressive strength (37.89%) of synthesized magnesium alloy hybrid composites has increased substantially because of heterogeneous deformation on the magnesium alloy matrix at the grain boundary.
- The corrosion-resistant magnesium alloy hybrid composites increased to a maximum of (37.81%) as a consequence of the continuous beta phase along the alpha grain boundary.

The limitation of this study is preferred range of B4C is up to 9 wt%. The experimental results showed that the highest contribution of reinforcement (9 wt. %) outperformed in properties enhancement of Mg–Al–Zn alloy. As a further investigation, it can be observed that various low-density reinforcements can significantly improve the properties of Mg–Al–Zn alloys. With lower fuel consumption and CO_2_ emissions, functional materials such as aluminium, zinc, and other alloying elements are added to pure magnesium to reduce environmental impact.

## Data Availability

The datasets used and analyzed during the current study are available from the corresponding author upon reasonable request.
